# Complete Genome Sequence of a Mixed-Subtype (H5N1 and H6N6) Avian Influenza Virus Isolated from a Duck in Hunan Province, China

**DOI:** 10.1128/genomeA.00310-14

**Published:** 2014-04-24

**Authors:** Zhihua Liu, Bing Xu, Quanjiao Chen, Ze Chen

**Affiliations:** a College of Life Sciences, Hunan Normal University, Changsha, Hunan, China; b Center for Emerging Infectious Diseases, Wuhan Institute of Virology, Chinese Academy of Sciences, Wuhan, Hubei, China; c Shanghai Institute of Biological Products, Shanghai, China; d Department of Environmental Science and Engineering, Tsinghua University, Beijing, China

## Abstract

We isolated an avian influenza virus, A/duck/Hunan/747/2011(mixed), which included the H5N1 and H6N6 subtypes, from a duck in China. Phylogenetic analysis indicates that the H5 hemagglutinin (HA) gene belongs to clade 2.3.2.1, the H6 HA gene belongs to the group II lineage, and the other internal genes show different recombination events.

## GENOME ANNOUNCEMENT

Highly pathogenic H5N1 avian influenza (HPAI) viruses have caused serious outbreaks in domestic chickens in multiple countries, including China (1), and they have posed a significant threat to public health in recent years. There are many disease events in humans that are associated with avian influenza virus (AIV)-infected poultry (2). Therefore, persistent monitoring of AIV in live poultry markets is important.

In December 2011, we isolated an AIV mixed-subtype virus from a duck in Hunan province. We sequenced the genome using next generation sequencing (NGS) (Illumina). The viral genome consisted of 10 single-stranded RNA segments, PB2, PB1, PA, H5, H6, NP, N1, N6, M, and NS, with 2,280, 2,274, 2,151, 1,707, 1,701, 1,497, 1,350, 1,413, 759, and 678 nucleotides, respectively. A clonal H5N1 virus was also recovered, but not a clonal H6N6 virus, so we named this virus A/duck/Hunan/747/2011(mixed), including two (H5 and H6) hemagglutinin (HA) and two (N1 and N6) neuraminidase (NA) subtypes (3). Data have demonstrated that up to 26% of the isolates show evidence of mixed-subtype infection and high rates of genome reassortment (4).

The hemagglutinin gene of the H5N1 genome encodes multiple basic amino acids adjacent to the cleavage site (RRRKR/G), which indicates high pathogenicity in poultry (5). The receptor-binding sites (RBS) of HA prefer to bind avian cell-surface receptors (6). The H6 HA receptor-binding sites (A138, E190, L194, G225, Q226, and G228; H3 numbering here and below) were not found to have alterations (7). The H6 HA gene cleavage site showed that this virus is a typical low-pathogenicity AIV. The stalk of the N1 protein at sites 49 to 68 has a 20-amino-acid deletion, which is considered to be necessary for virus adaptation to domestic fowl (8). The possible enhancement of virus virulence markers in PB2, such as E158G, E627K, or D701N, was not found (9, 10). However, a D92E substitution in the NS1 protein was found in the isolate and is considered to be involved in enhanced virus pathogenicity (11).

In the phylogenetic tree, all of the internal gene segments of the isolated virus were closely related to those from the avian H5N1 virus, except the M gene, which was closely related to that of the avian H6N6 virus.

This study demonstrated that mixed infections exist in nature and that they may promote virus recombination, thereby creating a novel virus. Strengthening epidemiological monitoring of influenza viruses is critical for improving understanding of the mechanism of AIV recombination and will provide relevant information to prevent and control avian influenza.

### Nucleotide sequence accession numbers.

The genome sequences of A/duck/Hunan/747/2011(mixed) have been deposited in GenBank under accession numbers KJ484606 through KJ484615.
